# *Hedyotis diffusa* plus* Scutellaria barbata* Induce Bladder Cancer Cell Apoptosis by Inhibiting Akt Signaling Pathway through Downregulating miR-155 Expression

**DOI:** 10.1155/2016/9174903

**Published:** 2016-02-17

**Authors:** Li-Tao Pan, Yip Sheung, Wen-Peng Guo, Zhi-Bin Rong, Zhi-Ming Cai

**Affiliations:** ^1^The Affiliated Clinical College Shenzhen Second People's Hospital, Anhui Medical University, Hefei 230032, China; ^2^The First Affiliated Hospital of Shenzhen University, Shenzhen, Guangdong 518035, China; ^3^The University of Hong Kong, Pokfulam, Hong Kong; ^4^Guang'anmen Hospital, China Academy of Chinese Medical Science, Beijing 100053, China

## Abstract

Traditional Chinese medicine is increasingly used to treat cancer. Our clinical experiences identify* Hedyotis diffusa* plus* Scutellaria barbata* as the most common herb-pair (couplet medicinal) used for the core treatment of bladder cancer. This study aims to investigate the antitumor effect of the herb-pair in bladder cancer cells. The results show that* Hedyotis diffusa* plus* Scutellaria barbata* inhibited bladder cancer cell growth and clone formation in a dose-dependent and time-dependent manner. It also induced cell apoptosis through decreasing Akt activation and reducing the expression of antiapoptotic proteins Bcl-2 and Mcl-1. Further experiments showed that miR-155 was reduced by the herb-pair and miRNA-155 inhibitor induced cell apoptosis and suppressed Akt activation. Overexpression of miR-155 reversed herb-pair induced cell apoptosis through activating Akt pathway in both bladder cancer cell lines. The findings reveal that* Hedyotis diffusa* plus* Scutellaria barbata* reduce Akt activation through reducing miR-155 expression, resulting in cell apoptosis. It demonstrated the potential mechanism of* Hedyotis diffusa* plus* Scutellaria barbata* for the core treatment of bladder cancer.

## 1. Background

Bladder cancer is one of the common cancers worldwide. The majority of bladder cancers are low-grade noninvasive tumors which may progress to the invasive phenotype. In contrast to noninvasive bladder cancer, muscle-invasive tumors tend to metastasize to other organs and have a very poor prognosis [1]. Several treatment strategies, including chemotherapy, radiotherapy, and combined radiochemotherapy, were used in the clinic and overall survival rate was improved. However, the survival benefits of these therapies are modest, and the factors affecting quality of life (QOL), such as fatigue, pain, morbidity due to lymphadenectomy, side effects from chemotherapy or radiotherapy, and menopausal symptoms, are still of considerable concern to patients [2, 3]. Thus, novel and more effective drugs or strategies need to be developed to improve the treatment and prognosis of bladder cancer patients.

Traditional Chinese medicine (TCM) has been widely used for treatment of bladder cancer patients, which is thought to be beneficial to patients' QOL and only has minimal side effect. TCM is reported to be effective by 86.7% of the patients as bladder cancer therapies that can improve the immune system, reduce the discomfort of chemotherapy and radiotherapy, and relieve menopausal symptoms [4]. However, the anticancer effects of TCM in bladder cancer have not been well studied.

For TCM prescriptions are usually complicated, the evaluation of the composition of TCM prescriptions for bladder cancer is crucial for determining the core TCM treatment. A core treatment is defined as the most commonly used TCM that is combined in a single prescription to form the major fraction of an herbal prescription for a specific disorder; therefore, each individual prescription includes the core treatment and modifications based on the patient's signs and symptoms. The core treatment of a specific disorder can only be identified by analyzing the patterns of TCM combinations [5–7]. Multiherb prescriptions of TCM often include special herb-pairs for mutual enhancement, assistance, and restraint. The term “herb-pair (also known as couplet medicinal)” refers to the use of two single herbs that are usually used together to treat a specific disease in order to enhance efficacy or minimize adverse effects [8, 9].

By analyzing a population-based TCM prescription database in our hospital for bladder cancer and literatures (Table 1) [10, 11],* Hedyotis diffusa* plus* Scutellaria barbata* was identified as the most common herb-pair used for the core treatment of bladder cancer. The aim of this study was to explore the antitumor effect and to investigate the detailed antitumor mechanisms of the herb-pair (*Hedyotis diffusa* plus* Scutellaria barbata*). To make the study easier, the main composite of the herb-pair was extracted and named as Hd-Sb for further investigating.

MicroRNAs (miRNAs) are small noncoding RNAs and are associated with cell proliferation, differentiation, apoptosis, and other biological processes [12]. Studies have showed that several flavonoids, such as luteolin [13, 14], naringin [15], and fisetin [16], regulate miRNA expressions to induce cell apoptosis, chondrosarcoma migration inhibition, and cancer cell growth arrest. These findings revealed that regulation of miRNA expression is a new mechanism of flavonoid-induced biological changes in cancer cells and provide a clue to investigate the possibility of miRNA changes induced by Hd-Sb extracted from herb-pair* Hedyotis diffusa* plus* Scutellaria barbata*.

In this study, we investigated the anticancer effect of* Hedyotis diffusa* plus* Scutellaria barbata* and related mechanisms in bladder cancer cells. Our results demonstrated that* Hedyotis diffusa* plus* Scutellaria barbata* inhibits bladder cancer cell growth and clone formation and induces cell apoptosis through inhibiting miR-155 expression and Akt pathway.

## 2. Methods

### 2.1. Cell Culture and Reagents

The human bladder cancer cell lines 5637 and T24 were purchased from the American Type Culture Collection (ATCC, Manassas, VA, USA). Both of them were maintained in RPMI-1640 medium supplemented with 10% heat-inactivated fetal bovine serum, glutamine, and antibiotics at 37°C in 5% CO_2_.

p-Akt and Akt antibodies were bought from Cell Signaling Technology (USA). Antibodies to *β*-actin, Bcl-2, mcl-1, and cleaved caspase-3, as well as horseradish peroxidase- (HRP-) linked anti-rabbit IgG, were purchased from Beyotime Institute of Biotechnology (China). MTT were purchased from Sigma (USA). MTT, Annexin-V-FITC, and PI were purchased from Beyotime Institute of Biotechnology, respectively. miR-155 mimics and inhibitor or negative siRNA control, as well as the primers for U6 and miR-155, was brought from Qiagen (Germany).

### 2.2. Herbal Extract Preparation Procedure

Dried powder samples of aqueous herbal extracts (*Hedyotis diffusa* and* Scutellaria barbata*) were purchased from Xian Changyue Ltd. (Xian, China). The herbal extract preparation was prepared by the following steps: first, the dried powder composition comprising* Hedyotis diffusa* and* Scutellaria barbata* (50 mg each) was dissolved and mixed into 5 mL of Milli Q water produced by Milli Q Synthesis A10 Water Purification System (EMD Millipore, Germany) at 70°C for 30 min under vortexing every 5 min; secondly, after cooling to room temperature, the insoluble materials were removed by centrifugation at 10,000 rpm by an Eppendorf 5424 microcentrifuge (Eppendorf AG, Germany) for 10 min; thirdly, the supernatant was recovered and sterilized by passage through a syringe filter with a 0.22 *μ*m (Pall, New York, USA) membrane; finally, the supernatants of herbal extracts were stored at −20°C for further use [17, 18].

### 2.3. Cell Viability Assay

The cell viability was measured by MTT cell viability assay. 1 × 10^4^ cells were planted in a 96-well plate and then incubated with different concentration of Hd-Sb for indicated times. MTT solution (0.5 mg/mL) was added and incubated for another 3 hours. 100 *μ*L of a solution containing 10% SDS and 0.01 M HCl was added to dissolve the crystals. The absorption at 570 nm (with 630 nm as the reference wavelength) was measured by Microplate ELISA Reader. Relative cell viability was determined as a fold of the control. All cell viability assays were performed in triplicate and repeated in 3 independent experiments.

### 2.4. Colony Formation Assay and Apoptosis Assay

500 5637 or T24 cells were seeded in 6-well plate and treated with Hd-Sb for 8 days. The colonies were fixed with methanol and stained with crystal violet. The number of colonies containing more than 50 cells was counted and presented as the fold of control. This experiment was performed in triplicate and repeated in 3 independent experiments.

5637 or T24 cells were harvested after treatment with Hd-Sb, followed by washing with PBS twice. Then the cells were stained with Annexin-V-FITC and PI in binding buffer for 30 min at room temperature. Analysis was performed on a BD FACSCanto flow cytometer (USA). Annexin-V and PI negative (Annexin-V^−^PI^−^) cells were living cells and the percentage of these cells was presented by histogram.

### 2.5. Western Blot Analysis

Cell lysates were separated by electrophoresis on polyacrylamide gels containing sodium dodecyl sulfate (SDS-PAGE) and then the proteins were transferred to polyvinylidene difluoride membranes. The membrane was blocked with 5% nonfat milk and then incubated with the primary antibody. After washing, the membrane was exposed to HRP-conjugated secondary antibody. The bands were visualized and captured using the ECL reagent (Beyotime, China) and X-ray films, respectively. The pixel densities of proteins were quantified using ImageJ software.

### 2.6. miRNA Isolation and Real-Time PCR

miRNA was isolated using miRNA pure Mini Kit (ComWin Biotechnology, China) according to the manufacturer's instructions. miScript II RT kit (Qiagen) was used for reverse transcription of miRNA. The expression of miR-155 was determined by miScript SYBR Green PCR Kit (Qiagen), using real-time PCR. miR-155 expression was normalized to the U6 based on their Ct values.

### 2.7. Transfection of miRNA Mimics or Inhibitor

5637 and T24 cells were transfected with miR-155 mimics (50 nM), anti-miR-155 (50 nM), or a negative control siRNA (50 nM) using the Lipofectamine 2000 Transfection Reagent (Invitrogen) according to the manufacturer's instructions.

### 2.8. Statistical and Data Analysis

The results were presented as means ± SD from three independent experiments. Statistical analysis was performed by Student's *t*-test or one-way ANOVA to determine the statistical significance for the experiments. A *P* value < 0.05 was regarded as statistically significant.

The support factor and confidence factor were used for association rule mining. Support factor refers to the ratio of coprescriptions of herbal A plus herbal B for all prescriptions, and the confidence factor is the ratio of coprescriptions of herbal A plus herbal B for all prescriptions including herbal A. In brief, the support factor was the prevalence of all TCM among all prescriptions, and the factors were used initially to rule out uncommon TCM. On the other hand, the confidence factors were used to determine the strength of connections between every two TCM combinations (herbal-pair) and were based on conditional probability.

## 3. Results

### 3.1. Hd-Sb Inhibits Bladder Cancer Cell Growth and Clone Formation

**Figure 1:**
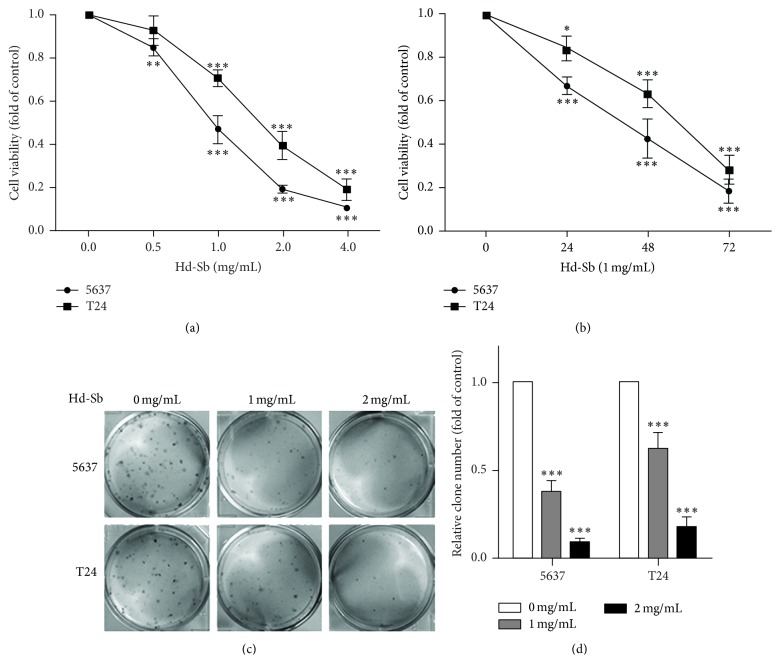
Hd-Sb inhibits bladder cancer cell growth and clone formation. (a) 5637 and T24 cells were treated with different concentration of Hd-Sb for 48 h and the cell viability was determined using MTT assay. (b) 5637 and T24 bladder cancer cells were treated with 1 mg/mL Hd-Sb for indicated times and the cell viability was measured. (c) 5637 and T24 cells were treated with 1 mg/mL or 2 mg/mL for 8 days and the clones were stained. (d) Stained clones were counted and presented as a fold of control by histogram. (The asterisks represent the value of *P*, where ^*∗*^ *P* < 0.05, ^*∗∗*^ *P* < 0.01, and ^*∗∗∗*^ *P* < 0.001.)

To investigate the anticancer activity of Hd-Sb in bladder cancer cells, we first measured the effect of Hd-Sb on cell growth. 5637 and T24 bladder cancer cells were treated with different concentration of Hd-Sb for 48 h and the cell viability was gradually reduced after treatment with higher dose of Hd-Sb (Figure 1(a)). 5637 cells were more sensitive to Hd-Sb compared with T24 cells. 1 mg/mL Hd-Sb induced further decrease of cell viability by about 80% after 72 h (Figure 1(b)). The inhibition of Hd-Sb on cell growth was also confirmed by using clone formation assay. Hd-Sb significantly reduced the clone formation of 5637 and T24 cells in a dose-dependent manner (Figures 1(c) and 1(d)). These results suggested that Hd-Sb inhibits bladder cancer cell growth, as well as clone formation.

### 3.2. Hd-Sb Induces Apoptosis of Bladder Cancer Cells

**Figure 2:**
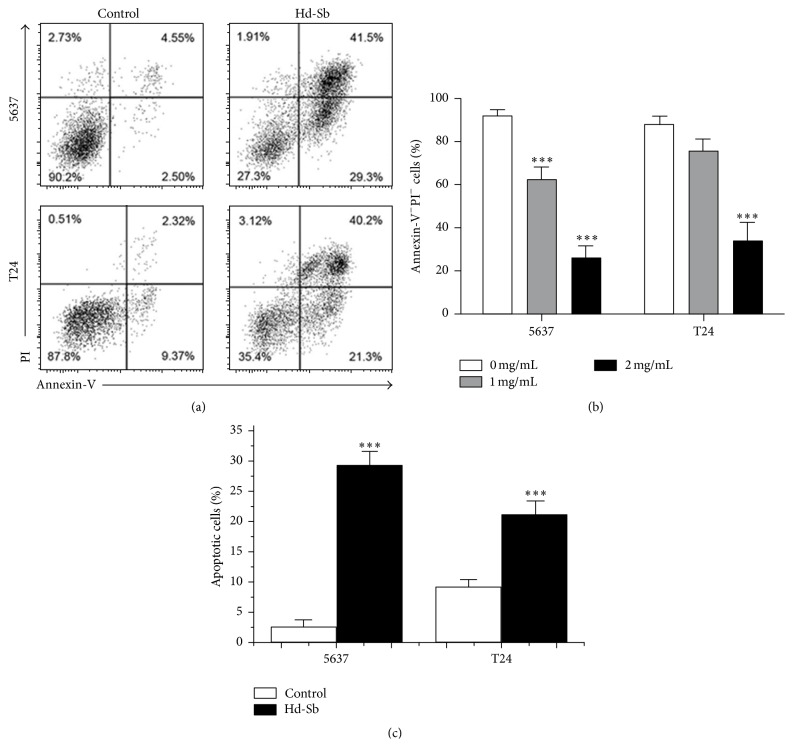
Hd-Sb induce bladder cancer cell apoptosis. (a) 5637 and T24 cells were treated with Hd-Sb for 48 h and the cell apoptosis was determined by Annexin-V and PI staining. One representative FACS profile is shown. (b) The percentage of living cells (Annexin-V^−^PI^−^) from three independent experiments was presented by bar. (c) Cell apoptosis induction was observed in Hd-Sb treated bladder cancer 5637 and T24 cells using flow cytometry analysis. (The asterisks represent the value of *P*, where ^*∗*^ *P* < 0.05, ^*∗∗*^ *P* < 0.01, and ^*∗∗∗*^ *P* < 0.001.)

We next investigated the effect of Hd-Sb on apoptosis in 5637 and T24 bladder cancer cells. The cells were treated with 1 mg/mL and 2 mg/mL Hd-Sb for 48 h and significant decrease of living cells was observed (Figures 2(a) and 2(b)). Less apoptosis was detected in T24 cells compared with that in 5637 cells when 1 mg/mL Hd-Sb was used, suggesting less sensitivity of T24 cells to Hd-Sb (Figure 2(c)).

### 3.3. Hd-Sb Suppresses Bcl-2, Mcl-1 Expression, and Activation of Akt

**Figure 3:**
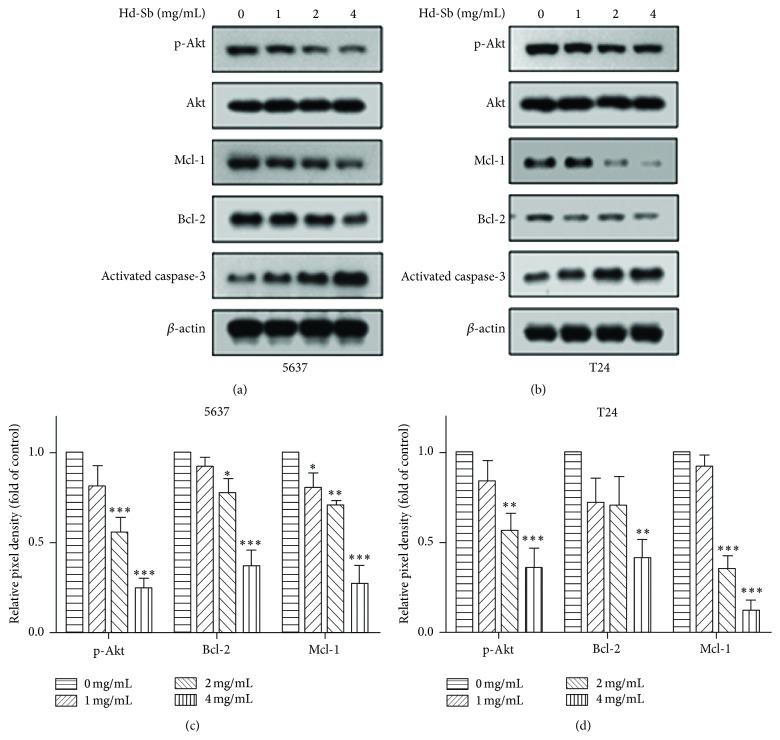
Hd-Sb reduce the activation of Akt pathway and the expression of antiapoptotic proteins. 5637 (a) and T24 (b) were treated with different concentration of Hd-Sb for 48 h and the proteins phosphorylated Akt, Akt, Mcl-2, Bcl-2, activated caspase-3, and *β*-actin were detected by Western blot. ((c) and (d)) The pixel density of these proteins was normalized to *β*-actin and the relative values from three independent experiments were presented by histogram. (The asterisks represent the value of *P*, where ^*∗*^ *P* < 0.05, ^*∗∗*^ *P* < 0.01, and ^*∗∗∗*^ *P* < 0.001.)

To study the molecular mechanisms of bladder cancer cell apoptosis induced by Hd-Sb, we next measured the expression levels of antiapoptosis proteins and the activation of Akt (Figures 3(a) and 3(b)). Hd-Sb inhibited the phosphorylation of Akt, while the total Akt was not changed. Meanwhile, expressions of Mcl-1 and Bcl-2 were significantly reduced by Hd-Sb. Hd-Sb also increased the activation of caspase-3, a key proapoptosis protein.

### 3.4. Hd-Sb Induce Cell Apoptosis through Inhibiting miR-155 Expression

**Figure 4:**
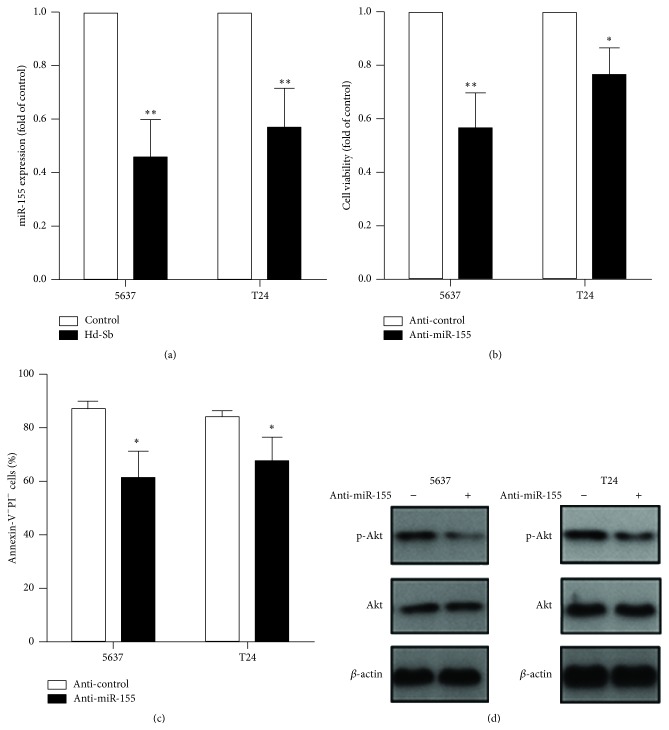
Hd-Sb decreases miR-155 expression and anti-miR-155 promotes cell apoptosis through Akt pathway. (a) 5637 and T24 cells were treated with 1 mg/mL Hd-Sb and the expression of miR-155 was determined using RT-PCR for 48 h. The cell viability (b) and cell apoptosis (c) were measured using MTT assay and FACS analysis. (d) 5637 and T24 cells were transfected with miR-155 inhibitor or control siRNA for 48 h and the phosphorylated Akt, Akt, and *β*-actin were detected using Western blot. (The asterisks represent the value of *P*, where ^*∗*^ *P* < 0.05, ^*∗∗*^ *P* < 0.01, and ^*∗∗∗*^ *P* < 0.001.)

Previous study has reported that miR-155 could regulate PI3K-Akt pathway to inhibit the cell viability and we also found the Akt pathway was inhibited by Hd-Sb. Therefore, we next investigated if miR-155 is associated with Hd-Sb-induced inhibition of Akt. After the treatment with Hd-Sb, the expression of miR-155 was significantly decreased in both 5637 and T24 cells (Figure 4(a)). After the transfection with miR-155 inhibitor in 5637 and T24 cells, the cell viability and the percentage of living cells were reduced (Figures 4(b) and 4(c)). Furthermore, miR-155 inhibitor reduced the phosphorylation of Akt, indicating a positive role of miR-155 on Akt pathway (Figure 4(d)). Together, these findings indicated that Hd-Sb could inhibit miR-155 expression and decreased miR-155 induces apoptosis through negatively regulating Akt pathway in bladder cancer cells.

### 3.5. miR-155 Inhibits Hd-Sb-Induced Cell Apoptosis through Increasing the Activation of Akt Pathway

**Figure 5:**
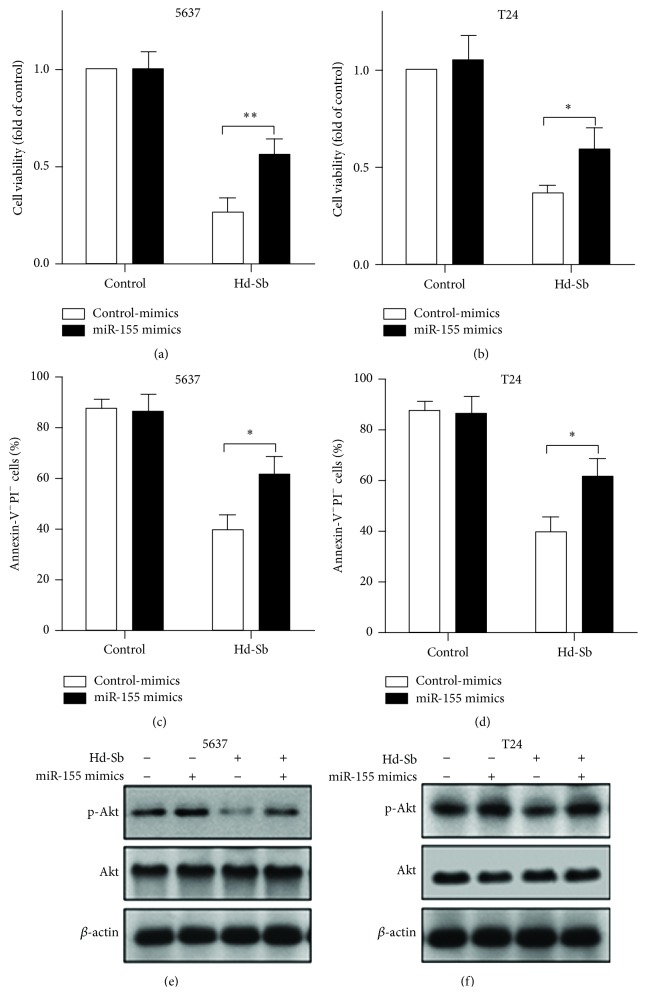
Higher miR-155 reverses Hd-Sb-induced cell apoptosis. 5637 (a) and T24 (b) cells were transfected with miR-155 mimics or control-mimics for 24 h and then treated with 1 mg/mL Hd-Sb for another 48 h. The cell viability was measured after the treatment. Cell apoptosis in 5637 (c) and T24 (d) cells was determined using FACS analysis and the percentage of Annexin-V^−^PI^−^ cells was presented by bar. Phosphorylated Akt, Akt, and *β*-actin in 5637 (e) and T24 (f) cells were detected by Western blot after the transfection and treatment. (The asterisks represent the value of *P*, where ^*∗*^ *P* < 0.05, ^*∗∗*^ *P* < 0.01, and ^*∗∗∗*^ *P* < 0.001.)

Due to the positive role of miR-155 on Akt activation and cell survival, we next investigated the roles of miR-155 on Hd-Sb-induced cell apoptosis. miR-155 mimics significantly suppressed the inhibition of cell viability induced by Hd-Sb in both 5637 and T24 cells (Figures 5(a) and 5(b)). Also, the survival of bladder cancer cells induced by Hd-Sb was increased by miR-155 (Figures 5(c) and 5(d)). Moreover, miR-155 increased phosphorylation of Akt in 5637 and T24 cells treated with Hd-Sb (Figures 5(e) and 5(f)). These results suggested that miR-155 could reverse Hd-Sb-induced cell apoptosis by promoting the activation of Akt pathway.

## 4. Discussion

Although several approved chemotherapeutic drugs are used in the treatment of bladder cancer patients, the toxic side effects and drug resistance are still the major problems in clinic. Therefore, the drugs with minimum side effect and maximum efficacy are wanted for cancer treatment.* Hedyotis diffusa* plus* Scutellaria barbata* is a natural anticancer herb-pair and has less toxicity in normal cells [4, 8]. However, the detailed anticancer effect of it in bladder cancer and the underlying mechanisms are not well known. In the present study, our results demonstrated the inhibition of bladder cancer cell growth by the herb-pair and revealed that it induces cell apoptosis through suppressing miR-155 expression and Akt pathway.

*Hedyotis diffusa* plus* Scutellaria barbata* induced apoptosis and cell cycle arrest in several cancer types are well investigated [4, 8, 19–22]. In addition, it is also reported that herb-pair suppresses cancer cell invasion and migration and could reverse multidrug resistance of leukemia cells [4, 23–25]. Our findings showed that water soluble extracts Hd-Sb from* Hedyotis diffusa* plus* Scutellaria barbata* inhibited cell growth and clone formation and induced apoptosis in two bladder cancer cell lines, and Hd-Sb induced apoptosis mainly through suppressing Akt activation. Akt pathway plays a positive role in cell survival and regulates the expression of many antiapoptotic proteins. Thus, Hd-Sb induces bladder cancer cell apoptosis by inhibiting Akt pathway. Previous studies have showed that* Scutellaria barbata* induce apoptosis by transcriptional suppression of Mcl-1 and by decreasing the expression of Bcl-2 [26, 27]. Downregulation of antiapoptotic proteins Bcl-2 and Mcl-1 by Hd-Sb in bladder cancer cells was also observed. Moreover, Hd-Sb increased activation of caspase-3 which leads to apoptosis.

Akt signaling pathway is directly or indirectly regulated by cytokines, kinases, and miRNAs [28, 29]. miR-155 has been reported to positively regulate Akt pathway by targeting p85*α* [30] or SH2 domain-containing inositol 5′-phosphatase 1 (SHIP1) [31, 32]. p85*α* and SHP1 are inhibitor of the PI3K/Akt signaling pathway and the inhibition of these proteins leads to the activation of Akt pathway [30, 32]. Here, we found that Hd-Sb reduced the expression of miR-155 and that the inhibitor of miR-155 suppressed Akt activation, suggesting that Hd-Sb inhibits Akt pathway, at least in part, through inhibiting miR-155 expression. Moreover, overexpression of miR-155 reversed Hd-Sb-induced apoptosis and inhibition of Akt activation in bladder cancer, confirming the involvement of miR-155 in Hd-Sb-induced apoptosis. These results indicated that Hd-Sb reduces miR-155 expression, followed by inhibition of Akt activation and induction of apoptosis in bladder cancer cells.

Taken together, our results demonstrated the anticancer effect of Hd-Sb in bladder cancer cells and that Hd-Sb induced apoptosis by inhibiting miR-155 expression and Akt pathways. Considering the less toxicity of Hd-Sb in normal cells and our findings, the active compounds Hd-Sb extracted from* Hedyotis diffusa* plus* Scutellaria barbata* will be further investigated in order to find a novel drug for treatment of bladder cancer.

## Figures and Tables

**Table 1:** The top five most common herbal-pairs used for bladder cancer treatment.

Rank	Herbal-pair	Support factor (%)	Confidence factor (%)	Number of prescriptions (%)
1	*Hedyotisdiffusa*; *Scutellaria barbata*	18.0	73.22	361 (18.0)^∧^
		N/A	N/A	178 (50.4)^#^
		N/A	N/A	157 (75.12)^*∗*^

2	*Poria cocos*; *Polyporus umbellatus*	13.8	54.12	277 (13.8)^∧^
		N/A	N/A	280 (79.35)^#^
		N/A	N/A	146 (69.86)^*∗*^

3	*Desmodium styracifolium*; *Lonicera japonica* Thunb.	11.7	60.30	235 (11.7)^∧^
		N/A	N/A	253 (71.59)^#^
		N/A	N/A	113 (54.07)^*∗*^

4	Herba Lophatheri; liquorice root	10.0	58.92	202 (10.0)^∧^
		N/A	N/A	196 (55.42)^#^
		N/A	N/A	104 (49.76)^*∗*^

5	*Atractylodes*; Rhizoma Alismatis	8.8	43.32	178 (8.8)^∧^
		N/A	N/A	121 (34.04)^#^
		N/A	N/A	5 (2.39)^*∗*^

Note: the total number of prescriptions: ^∧^2004; ^#^354 [10]; ^*∗*^209 [11]. N/A: not available. The association rules were analyzed by software SPSS Modeler 14.1 (Apriori).

## Abbreviations

TCM: Traditional Chinese medicine
Hd-Sb: * Hedyotis diffusa* plus* Scutellaria barbata*
HRP: Horseradish peroxidase
miR-155: MicroRNA 155
QOL: Quality of life.
